# Red cell distribution width as a significant indicator of medication and prognosis in type 2 diabetic patients

**DOI:** 10.1038/s41598-017-02904-9

**Published:** 2017-06-02

**Authors:** Xiao-fen Xiong, Yuan Yang, Xianghui Chen, Xuejing Zhu, Chun Hu, Yachun Han, Li Zhao, Fuyou Liu, Lin Sun

**Affiliations:** Department of Nephrology, The Second Xiangya Hospital, Central South University, Changsha, Hunan 410011 China

## Abstract

Whether red cell distribution width (RDW) can be a potential indicator for diabetic nephropathy (DN) is unknown. A total of 809 type 2 diabetes mellitus (T2D) patients were divided into 4 groups according to the quartiles (Q) of the RDW (%): Q1 ≤ 12.4 (n = 229), 12.4 < Q2 ≤ 12.9 (n = 202), 12.9 < Q3 < 13.5 (n = 168), Q4 ≥ 13.5 (n = 210). Results showed that the levels in Q4 group was higher in age, disease duration, systolic blood pressure, blood urea nitrogen, creatinine, uric acid and proteinuria but lower in hemoglobin, serum albumin and glycosylated hemoglobin compared to Q1 group. Furthermore, the incidences of DN, diabetic peripheral neuropathy, hypertension and coronary heart disease in the Q3 or Q4 group were higher compared to Q1 group. Medications including calcium channel blockers and antiplatelet therapy also showed higher frequencies in Q3 or Q4 group compared to Q1. Logistic regression indicated that the antiplatelet therapy (OR = 2.065), hypertension (OR = 2.819), creatinine (OR = 4.473) and proteinuria (OR = 2.085) were positively associated with level of Q4 group, but higher hemoglobin (OR = 0.021) and serum Ca^2+^ (OR = 0.178) were negatively associated with Q4. This data suggest that high level of RDW in T2D patients indicates a higher risk and a poor prognosis for DN.

## Introduction

Diabetes mellitus (DM) is a metabolic disorder caused either by the insufficient production of insulin in islet cells of the pancreas or by resistance against secreted insulin in tissues, leading to an elevation in the glucose concentration in the blood. In China, a national survey in 2010 showed a DM prevalence of 9.65%, with the total number of DM patients up to 92.4 million and accounting for a quarter of worldwide DM patients in the population aged 20 to 79 years. Several studies have shown that plasma cholesterol levels, blood pressure, microalbuminuria and hyperglycemia are closely associated with the progression of DM^1, 2^. Additionally, recent studies have revealed serum uric acid level, carboxy-terminal propeptide and retinal venular diameter as significant indicators of diabetic complications such as diabetic nephropathy or retinopathy^3–5^. RDW, defined as the heterogeneity of circulating erythrocytes (anisocytosis), was used to distinguish the variable pathogenesis of anemia together with the MPV^6^. Malnutrition, including Fe deficiency and lack of vitamin B_12_ and folic acid, generates elevated RDW^7^. Recent studies have demonstrated that RDW may also be an effective predictor of morbidity and mortality in various diseases such as PH^8, 9^, NAFLD^10^, CHD^11–15^, stoke^16^, atherosclerosis^17^, prevalent dementia^18^, IBD^19^, ESRD^20^ and heart failure^21^. A study following 8175 adults for up to 6 years showed that the measurement of RDW may be used to predict mortality in CVD, cancer and other diseases^22^ in the early stages. Moreover, in a 5.5-year follow-up of 13039 patients diagnosed with PAD, the 1% increase of RDW was accompanied by the increased 10% in all-cause mortality. Also, RDW was considered as a prognostic marker in PAD patients^23^, which was extraordinarily higher in metabolic syndrome (MS) patients compared to those without MS^24^.

Recently, some studies have shown that increased RDW is associated with the incidence of DM^6, 25–27^. However, the explicit relationship between RDW and the basic indexes, drug treatment and related complications (such as chronic heart disease and diabetic retinopathy) remains implicit in T2D patients. Here, we study the characteristics of RDW and its association with distributions of clinical indexes in T2D patients.

## Results

### General characteristics of Q1 to Q4 in type 2 diabetic patients

**Figure 1:**
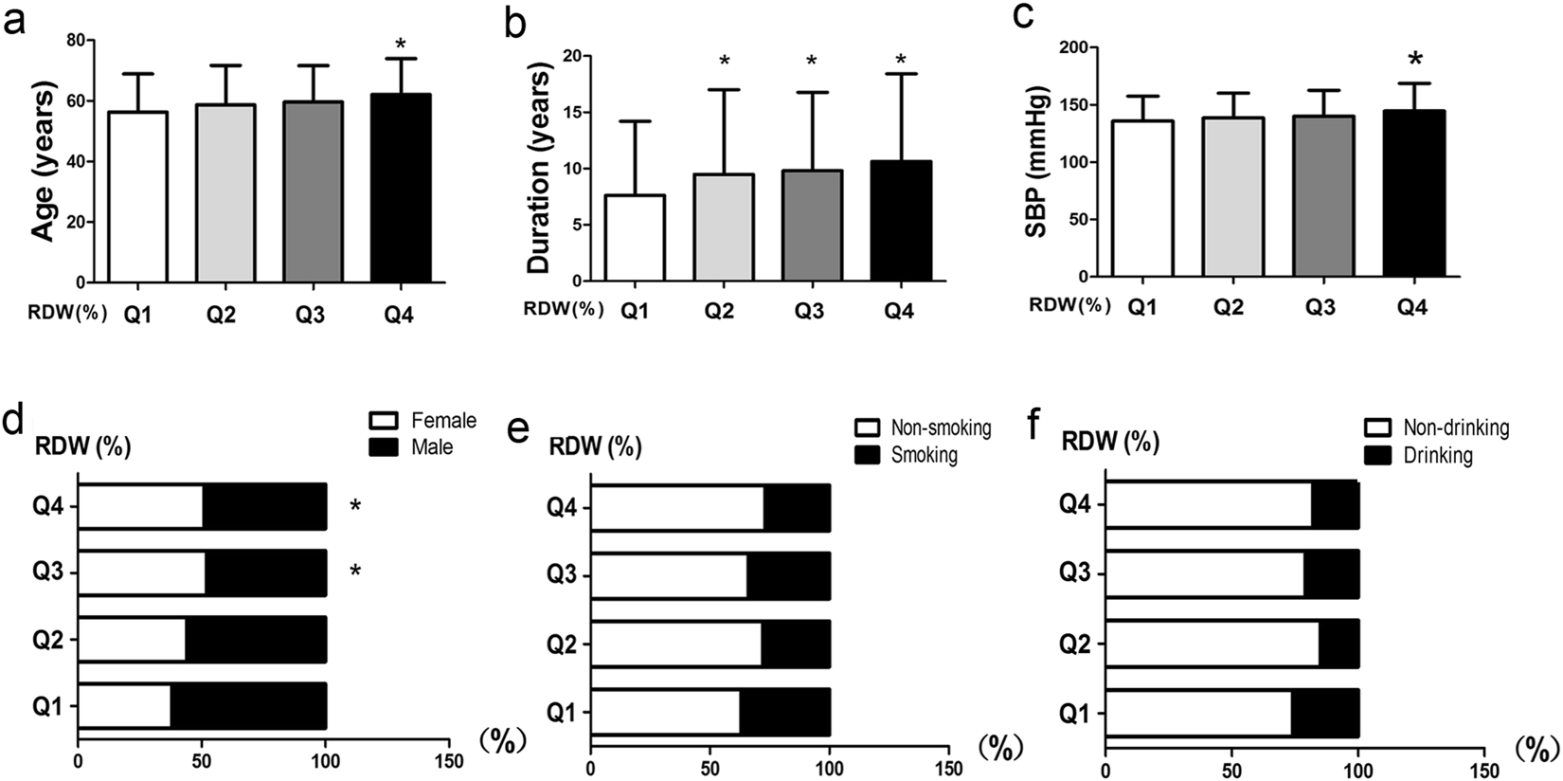
General characteristics of Q1 to Q4 in the T2D patients. (**a**) Distribution of age in Q1 to Q4; (**b**) Distribution of duration in Q1 to Q4; (**c**) Distribution of SBP in Q1 to Q4; (**d**) Distribution of male proportion in Q1 to Q4; (**e**) Distribution of smoking proportion in Q1 to Q4; (**f**) Distribution of drinking proportion in Q1 to Q4. *P value of less than 0.05 compared with the Q1. *Abbreviation:* SBP, systolic blood pressure.

As shown in Table 1 and in Fig. 1a–f, group Q4 showed the higher average age (62.10 ± 11.88 years) compared to Q1 group (56.33 ± 12.59 years), Fig. 1a. The male ratio was lower in the Q3 group (48.2%) compared to that of the Q1 group (62.0%), Fig. 1d. Moreover, compared to the Q1 group (7.63 ± 6.58), T2D patients of Q4, Q3 and Q2 group showed all the longer average duration years (10.65 ± 7.77, 10.24 ± 6.80, 10.03 ± 7.37), Fig. 1b, and higher average SBP (144.56 ± 24.18), Fig. 1c, and the proportion of smokers and drinkers (Fig. 1e, Fig. 1f). There were no significant differences in the distributions of DBP, BMI among the Q1 to Q4 groups.

**Table 1:** Distribution of basic characteristics from Q1 to Q4 in T2D patients.

Variable	Q1 (n = 229)	Q2 (n = 202)	Q3 (n = 168)	Q4 (n = 210)
Age (years)	56.33 ± 12.59	58.68 ± 13.04	59.64 ± 12.00	62.10 ± 11.88^*^
Male (%)	142 (62%)	113 (55.9%)	81 (48.2%)^*^	104 (49.5%)^*^
Smoking (%)	80 (36.7%)	57 (28.2%)	57 (33.9%)	54 (26.6%)
Drinking (%)	58 (26.5%)	30 (14.9%)	36 (21.4%)	37 (18.2%)
Duration (years)	7.63 ± 6.58	10.03 ± 7.37^*^	10.24 ± 6.80^*^	10.65 ± 7.77^*^
SBP (mmHg)	135.90 ± 21.71	138.62 ± 21.70	140 ± 22.83	144.56 ± 24.18^*^
DBP (mmHg)^a^	81.54 ± 10.96	82.32 ± 13.12	80.42 ± 13.68	79.44 ± 12.43
BMI (kg/m^2^)	25.44 ± 13.83	24. 67 ± 3.55	24.89 ± 3.23	24.30 ± 3.43

Notes: The analysis of variance (ANOVA) was used to compare the distribution of the variables when follows normal distribution, if not, the non-parametric Kruskal-Wallis test was applied; χ^2^ test was used to compare the difference of qualitative variables; ^a^Indicates the variables did not follow normal distribution. *Statistical *P* value < 0.05 compared to Q1. *Abbreviation*: SBP, systolic blood pressure; DBP, diastolic blood pressure; BMI, body mass index.

### Laboratory results of blood and urine in type 2 diabetic patients

**Figure 2:**
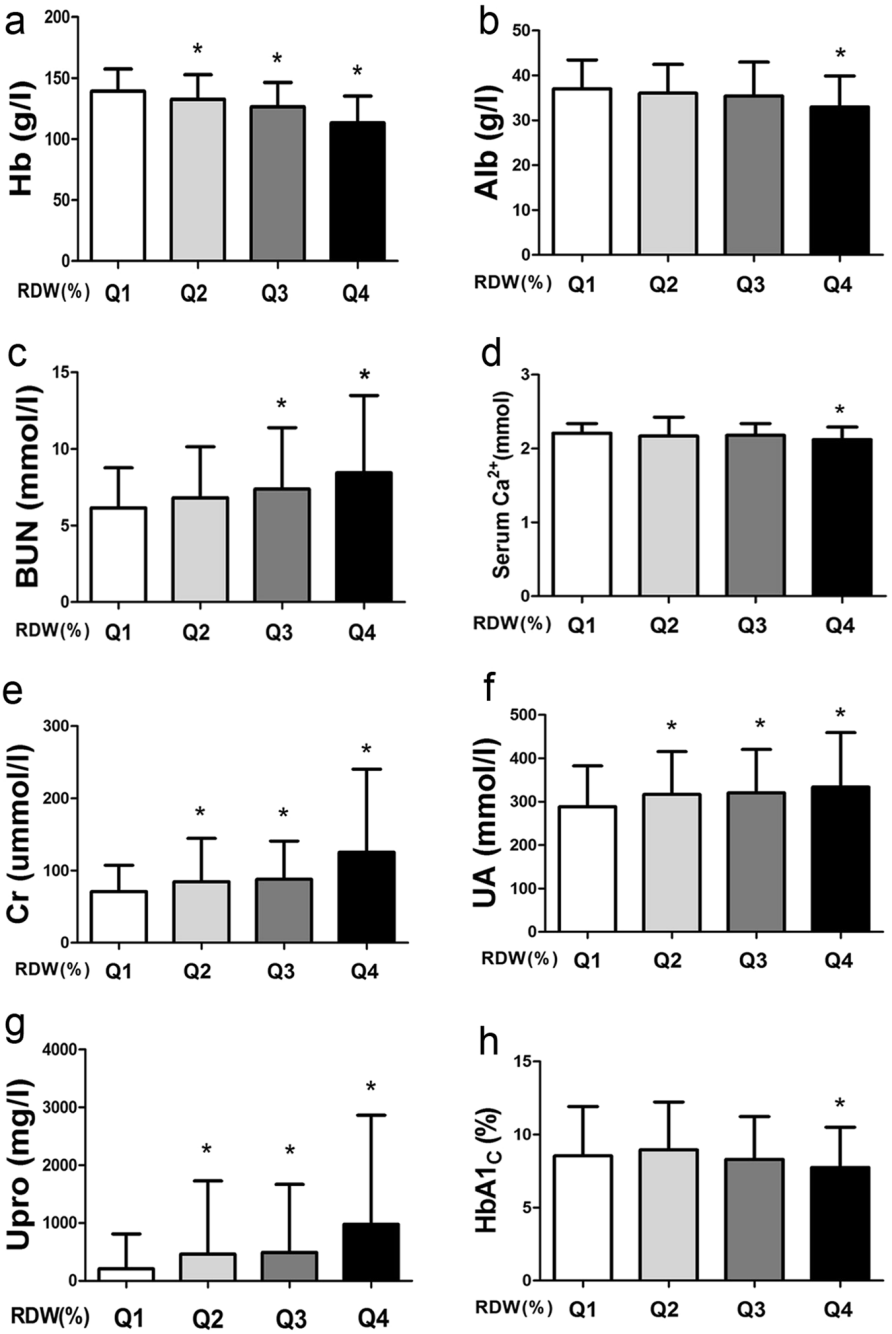
Laboratory results of blood and urine in T2D patients. (**a**) Distribution of Hb levels in Q1 to Q4; (**b**) Distribution of Alb levels in Q1 to Q4; (**c**) Distribution of BUN levels in Q1 to Q4; (**d**) Distribution of serum Ca^2+^ levels in Q1 to Q4; (**e**) Distribution of Cr levels in Q1 to Q4; (**f**) Distribution of UA levels in Q1 to Q4; (**g**) Distribution of Upro levels in Q1 to Q4; (**h**) Distribution of HbA1c levels in Q1 to Q4. *P value less than 0.05 compared with the Q1. *Abbreviation:* Hb, hemoglobin; Alb, serum albumin; BUN, blood urea nitrogen; Serum Ca^2+^: Serum calcium; Cr, serum creatinine; UA, uric acid; Upro, urinary protein excretion; HbA1c, glycosylated hemoglobin.

Compared to the Q1 group, in the Q4 group, the T-test or the non-parametric Kruskal-Wallis test showed that Hb (g/dL), Alb (g/L) and HbA1c (%) levels were significantly lower, but BUN (mmol/L), serum Ca^2+^ (mmol/L), Cr (μmmol/L), UA (mmol/L), Upro (mg/L) and HbA1c (%) levels were significantly higher (Table 2 and Fig. 2a–h) (P < 0.01). For the distributions of WCC (*10^9^), MPV (fl), PCT (%), P-LCR (%), PDW(%), TG (mmol/L), TC (mmol/L), HDL (mmol/L), LDL (mmol/L), serum phosphorus (mmol/L), and FBG (mmol/L), no significant differences were observed among the four groups.

**Table 2:** Blood and urine examinations of Q1 to Q4 in T2D patients.

Variable	Q1 (n = 229)	Q2 (n = 202)	Q3 (n = 168)	Q4 (n = 210)
Hb (g/dL)	139.26 ± 18.20	132.61 ± 20.15^*^	126.42 ± 19.92^*^	113.32 ± 21.90^*^
WCC (×10^9^)^a^	7.14 ± 2.13	6.97 ± 2.04	7.07 ± 2.14	7.34 ± 3.0
MPV (fL)^a^	11.33 ± 1.10	11.43 ± 1.23	11.99 ± 7.97	11.12 ± 1.28
PCT (%)^a^	0.23 ± 0.07	0.27 ± 0.06	0.24 ± 0.07	0.24 ± 0.12
P-LCR (%)	35.75 ± 8.75	36.59 ± 9.67	35.79 ± 9.05	34.17 ± 10.01
PDW (%)	14.35 ± 2.83	14.75 ± 3.30	14.40 ± 2.95	14.05 ± 3.25
Alb (g/L)^a^	37.53 ± 4.87	36.41 ± 5.37	36.29 ± 5.19	33.47 ± 5.67^*^
TG (mmol/L)^a^	2.26 ± 2.48	2.44 ± 2.63	2.60 ± 2.70	2.30 ± 3.35
TC (mmol/L)^a^	4.48 ± 1.25	4.55 ± 1.25	4.66 ± 1.26	4.35 ± 1.37
HDL (mmol/L)	1.01 ± 0.31	1.00 ± 0.26	1.03 ± 0.27	1.04 ± 0.35
LDL (mmol/L)	2.76 ± 0.91	2.80 ± 0.96	2.82 ± 1.02	2.63 ± 1.06
BUN (mmol/L)^a^	6.15 ± 2.62	6.89 ± 3.27	7.42 ± 3.99^*^	8.53 ± 5.00^*^
Cr (µmmol/L)^a^	71.06 ± 36.40	85.03 ± 59.86^*^	88.46 ± 52.63^*^	126.61 ± 114.93^*^
UA (mmol/L)	293.74 ± 86.54	318.62 ± 96.05^*^	322.54 ± 97.18^*^	338.62 ± 119.86^*^
Upro (mg/L)^a^	209.92 + 103.18	462.06 ± 126.51^*^	490.51 ± 117.93^*^	978.06 + 188.56^*^
Serum Ca^2+^ (mmol/L)	2.21 ± 0.13	2.19 ± 0.14	2.18 ± 0.16	2.12 ± 0.17^*^
Serum P (mmol/L)	1.07 ± 0.24	1.07 ± 0.25	1.10 ± 0.23	1.06 ± 0.26
FBG (mmol/L)^a^	8.34 ± 2.74	8.42 ± 2.83	8.07 ± 2.5	8.12 ± 3.05
HbA1c (%)^a^	9.27 ± 2.37	9.43 ± 2.62	8.83 ± 2.11	8.18 ± 2.11^*^

Notes: T-test for comparing the difference from Q1 to Q4; ^a^The distribution of the variables did not follow normal distribution, so the non-parametric Kruskal-Wallis test was used; *P < 0.05 compared to Q1 group. *Abbreviation*: Hb, hemoglobin; MPV, mean platelet volume; HbA1c, glycosylated hemoglobin; WCC, white cell count; MPV, mean platelet volume; PCT, plateletcrit; P-LCR, plate–large cell ratio; PDW, platelet distribution width. Alb, serum albumin; TG, triglyceride; TC, total cholesterol; HDL, high density lipoprotein; LDL, low density lipoprotein; BUN, blood urea nitrogen; Cr, serum creatinine; UA, uric acid; Upro, urinary protein excretion; FBG, fasting blood glucose.

### Complications and medications of the Q1 to Q4 groups in type 2 diabetic patients

**Figure 3:**
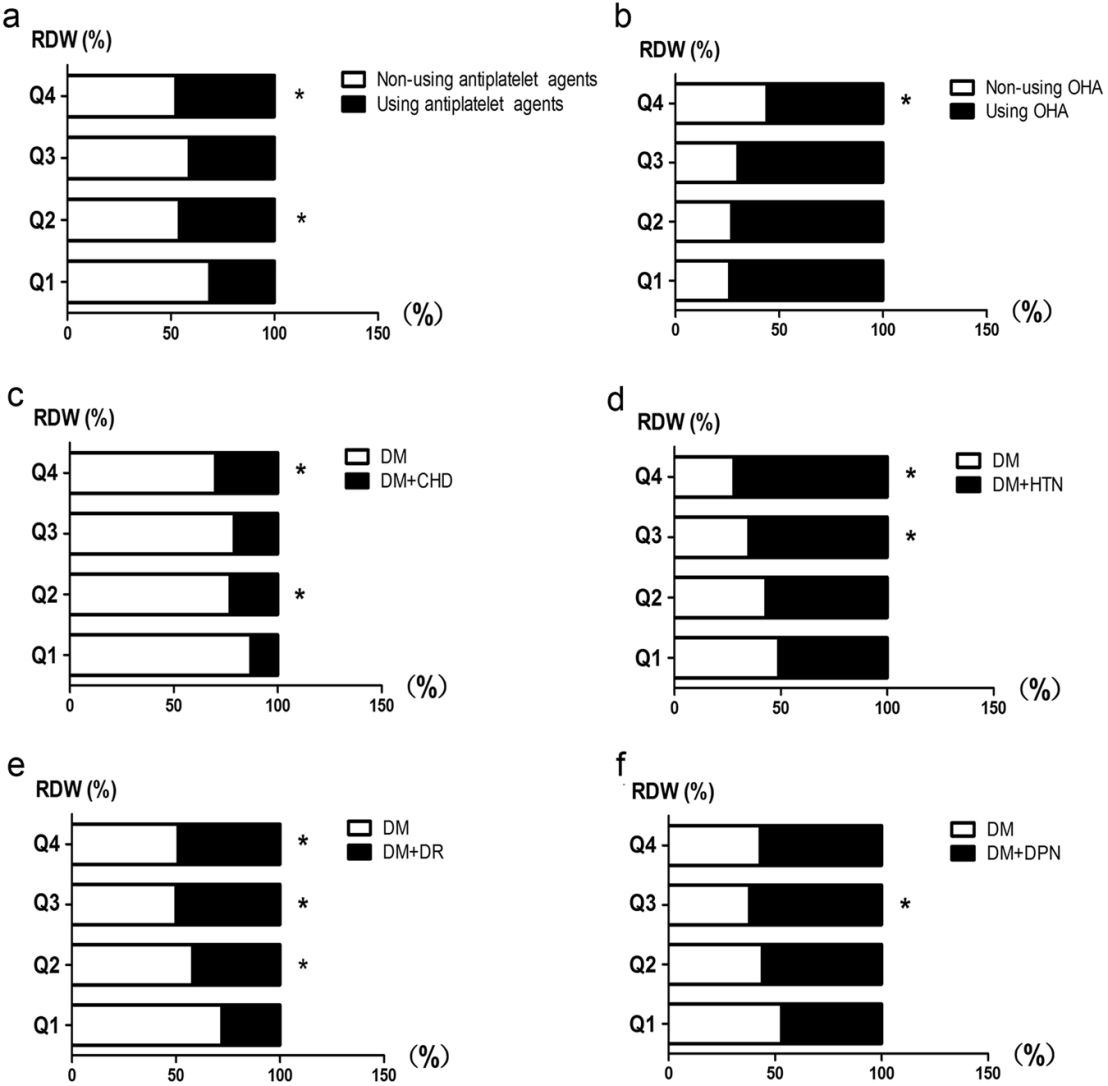
Distribution of Q1 to Q4 in complications and medications of T2D patients. (**a**) Proportion of using antiplatelet agents in Q1 to Q4; (**b**) Proportion of using OHA in Q1 to Q4; (**c**) Proportion of DM plus CHD in Q1 to Q4; (**d**) Proportion of DM plus HTN in Q1 to Q4; (**e**) Proportion of DM plus DR in Q1 to Q4; (**f**) Proportion of DM plus DPN in Q1 to Q4. *P value of less than 0.05 compared with the Q1. *Abbreviation:* OHA, oral hypoglycemic agent; DM, Diabetes mellitus; CHD, coronary heart disease; HTN, hypertension; DR, diabetic retinopathy; DPN, diabetic peripheral neuropathy.

Compared to the Q1 group, the patients in the higher Q3 or Q4 groups had increasing morbidities of DR, HTN and using CCB. The Q3 group had a higher rate of DPN compared to the Q1 group (61.9% vs 46.7%, *P* < 0.05). Moreover, groups Q2 and Q4 had more morbidities of CHD compared to the Q1 group (*P* < 0.05), and Q4 group used less OHA than Q1 group (*P* < 0.05). (Table 3 and Fig. 3a–f). No significant differences were found in either category of patient treatment, with or without ACE-I or ARB, β-Blocker, lipid-lowering agents, or insulin (*P* > 0.05).

**Table 3:** Complications and medications of Q1 to Q4 in T2D patients.

Variable	Q1 (n = 229)	Q2 (n = 202)	Q3 (n = 168)	Q4 (n = 210)
DR (%)	64 (27.9%)	85 (42.1%)^*^	84 (50%)^*^	103 (49%)^*^
DPN (%)	107 (46.7%)	114 (56.4%)	104 (61.9%)^*^	119 (56.9%)
HTN (%)	117 (51.1%)	116 (57.4%)	109 (64.9%)^*^	151 (71.9%)^*^
CHD (%)	31 (13.5%)	47 (23.3%)^*^	36 (21.4%)	64 (30.5%)^*^
ACE –I or ARB (%)	90 (39.3%)	95 (47.0%)	77 (45.8%)	97 (46.2%)
β-Blocker (%)	27 (11.8%)	35 (17.3%)	32 (19.0%)	33 (15.7%)
CCB (%)	60 (26.2%)	60 (29.7%)	66 (39.3%)^*^	90 (42.9%)^*^
Antiplatelet agents (%)	72 (31.4%)	93 (46.0%)^*^	69 (41.1%)	100 (47.6%)^*^
Lipid-lowering agents (%)	156 (68.1%)	148 (73.3%)	118 (70.2%)	129 (61.4%)
OHA (%)	169 (73.8%)	148 (73.3%)	117 (69.6%)	117 (55.7%)^*^
Insulin therapy (%)	157 (68.6%)	138 (68.3%)	124 (73.8%)	154 (73.3%)

Notes: *P value of χ^2^ test <0.05 compared to Q1 group. *Abbreviation*: DR, diabetic retinopathy; DPN, diabetic peripheral neuropathy; HTN, hypertension; CHD, coronary heart disease; ACE-I or ARB, use of an angiotensin-converting enzyme inhibitor or angiotensin II type I receptor blocker, respectively; CCB, calcium channel blocker; OHA, oral hypoglycemic agent; Insulin therapy, treatment with insulin including basal supported oral therapy.

### Binary Logistic regression models for RDW

As shown in Table 4, Q4 and Q1 of RDW were defined as the dependent variables in the Binary Logistic regression model. After adjustments for age and gender as confounding factors, the results showed a positive association between Q4 of RDW and antiplatelet therapy (OR = 2.065, 95% CI: 1.14–3.75), HTN (OR = 2.819, 95% CI: 1.49–5.35), Cr (OR = 4.473, 95% CI: 1.80–11.10), Upro (OR = 2.085, 95% CI: 1.19–3.67) and disease duration ≥10 years (OR = 3.189, 95% CI: 1.35–7.53). However, Hb ≥110 g/dL (OR = 0.021, 95% CI: 0.01–0.10) and the levels of serum Ca^2+^ ≥2.03 mmol/L (OR = 0.178, 95% CI: 0.08–0.39) were negatively associated with Q4 of RDW. For the Logistic regression model of Q3 vs Q1 of RDW, the number of significant variables in the model decreased from 7 to 4, including antiplatelet therapy (OR = 1.855), Cr (OR = 3.756), Upro (OR = 1.814) and disease duration ≥10 years (OR = 1.996). Moreover, in the Logistic regression model of Q2 vs Q1 of RDW, the results showed that the least significant variables were antiplatelet therapy (OR = 1.775) and Upro (OR = 1.569).

**Table 4:** Logistic regression model for RDW in T2D patients.

Variables	B	S.E.	Wals	N-OR	A-OR	95% Cl
Logistic regression model 1: Q4 vs Q1 as dependent variables						
Antiplatelet agents	0.725	0.304	5.697	2.127	2.065^*^	1.14–3.75
HTN	1.036	0.327	10.04	2.783	2.819^*^	1.49–5.35
Age^a^	0.268	0.134	3.982	1.432	1.307	1.01–1.70
Gender^a^	−0.140	0.293	0.229	1.807	0.869	0.49–1.54
Hb(2)^b^	−3.857	0.807	22.817	0.023	0.021^*^	0.01–0.10
Cr	1.498	0.464	10.438	4.617	4.473^*^	1.80–11.10
Serum Ca^2+^	−1.725	0.403	18.352	0.189	0.178^*^	0.08–0.39
Upro	0.735	0.288	6.491	2.075	2.085^*^	1.19–3.67
Duration(3)^b^	1.160	0.438	7.009	4.136	3.189^*^	1.35–7.53
Constant	−0.172	1.400	0.015	0.989	0.842	
**Logistic regression model 2: Q3 vs Q1 as dependent variables**						
Antiplatelet agents	0.618	0.241	6.597	1.820	1.855^*^	1.16–2.97
Cr	1.323	0.446	8.821	3.549	3.756^*^	1.57–8.99
Upro	0.596	0.234	6.484	1.713	1.814^*^	1.15–2.87
Duration(2)^b^	0.691	0.342	4.082	2.491	1.996^*^	1.02–3.90
**Logistic regression model 3: Q2 vs Q1 as dependent variables**						
Antiplatelet agents	0.574	0.220	6.772	1.769	1.775^*^	1.15–2.73
Upro	0.450	0.218	4.275	1.564	1.569^*^	1.02–2.40

Notes: ^a^Age and gender were considered confounding factors that were adjusted for in the logistic model; ^b^Dummy variables were set due to the characteristic of multi-classification in Hb and Duration; Hb (2) indicates a higher level of Hb (≥110 g/dL); Duration (2) indicates a duration of 3 to 9 years; Duration (3) indicates a duration ≥10 years; Wals, Wals’ χ^2^ test; N-OR, Non-adjusted OR (Odds ratio) value; A-OR, Adjusted OR value; 95% Cl, 95% confidence interval of A-OR; *P < 0.05 for Wals’ χ^2^ test.

## Discussion

Recently, the increasing prevalence of DM has become a global health problem. Disclosed risk factors include age, family history of diabetes, obesity, hypertension and high triglycerides^28^. In China, the crude and age-standardized prevalence of DM are 12.19% and 6.98%, respectively^29^. Several studies have focused on prognostic biomarkers that could indicate the incidences of coronary artery spasm (CAS), acute ischemic stroke (AIS), cardiovascular disease (CVD) and diabetic nephropathy (DN) in DM patients. Microalbuminuria is a remarkable biomarker for the diagnosis of DN^30^. Inflammatory biomarkers including WBC, TNF-α, matrix metalloproteinases (MMT) and dysglycemia are expressed concurrently to fit with the early stages of CVD in patients with diabetes^31^. Low high-sensitivity C-reactive protein (hs-CRP) levels in patients with DM are associated with a high risk of CAS^32^. The higher copeptin levels in the upper inter-quartile group (Q4 > 17.1 pmol/L) were associated with a higher death risk in short-term stroke prognosis in patients with T2D and stroke^33^. Currently, the significant indicators involved in the development or the prognosis of DM are still not fully understood.

In this study, we demonstrated that changes in RDW are associated with T2D. Disease duration and SBP showed positive associations with the higher Q4 group of RDW, but other clinical indexes, including serum Ca^2+^, Hb or HDL, were negatively associated with the Q4 group of RDW. One retrospective study^22^ indicated that RDW is an age-associated biomarker in people >45 years old. Here, we also found that elderly patients had significantly higher Q4 values of RDW than younger patients, *P* < 0.05 (Fig. 1a). Furthermore, the regression model showed that age was positively associated with RDW (Table 4). One possible explanation involves the greater potential of seniors to be in a state of inflammation, nutritional deficiency and other complications. Another retrospective study^6^ that included 260 T2D patients and 44 healthy control subjects found that RDW was correlated with BMI. However, our study showed that the changes of RDW value were not significantly associated with the BMI levels in diabetic patients. Additionally, in the present study, SBP in the Q4 group was obviously higher compared to the Q1 group (Fig. 1c) and HTN was positively associated with RDW as detected by the regression model (Table 4), which was consistent with reports by Dada, O. A. *et al*.^34^. However, Malandrino N’s study indicated that the duration of DM had no significant association with RDW, which contradicts our study that indicates that the higher levels of RDW are positively associated with the longer duration in T2D patients (Table 4 and Fig. 1b)^35^.

The levels of RDW were measured in 26709 non-diabetic subjects over a 14-year follow-up period in a report by Engstrom G, showing that RDW was positively correlated to HbA1c, indicating that the HbA1c would increase by 0.10% per Standard Deviation (SD) elevation in RDW^26^. In our study, the HbA1c in the Q4 group showed a significant decrease compared to Q1 group (Fig. 2h). Recently, Lippi G *et al*.^36^ showed a negative correlation between RDW and Hb in 4874 outpatients, which was consistent with our results (Fig. 2a). Additionally, a negative association was identified between RDW and HDL-cholesterol in the multivariate Logistic regression after adjustment for age, Hb, and MCV, which was different from our study, which showed no obvious discrepancy between HDL and RDW. The reason may be related to the fact that the patients in our study were receiving appropriate treatment, including control of lipidemia, because all indicators such as TG, TC and LDL in lipidemia showed no differences (*P* > 0.05). On the other hand, we also concluded that serum Ca^2+^ was negatively associated RDW since the lower Q1 of RDW showed the higher level of serum Ca^2+^ (Table 2). A possible explanation is that higher serum Ca^2+^ may increase the deformability of red cells, leading to reduced RDW.

For the relationship between RDW and proteinuria, the results of several studies showed the consistence with our study. For example, Zhang M *et al*.^27^ assessed 320 patients who were newly diagnosed with T2D and indicated that RDW was a risk factor for microalbuminuria (MAU), with a value of 0.79 for the area under the curve as compared to a healthy group. In addition, Caroline J *et al*.^37^ collected 196 T2D patients with DN (57%), diabetic neuropathy (46%) and peripheral arterial disease (26%) and found that RDW level is a high risk factor in DN (OR: 1.64, 95% CI: 1.15–2.35). Similarly, our results indicate that RDW is positively associated with proteinuria (Table 4) after adjustment of the potential confounders such as age and gender.

Furthermore, one study of 786 older women suggested that a higher quartile of RDW tended to be associated with a higher interleukin-6 level^38^. It was also reported that TNF-α and interleukin-6, which reflect pro-inflammatory conditions in DM patients, have significantly close relationships with proteinuria^39^. Moreover, chronic inflammatory cytokines displayed a key role in damaging and increasing the permeability of glomerular endothelial cells, resulting in proteinuria^40^. Therefore, proteinuria could be used to reflect the level of inflammation, providing a reasonable explanation for the close association between RDW and proteinuria. Interestingly, proteinuria was significantly related to oxidative stress, which is involved in the oxidation of the LDL fatty acids, and it was also associated with RBC fragments^41, 42^ that give rise to incremental increases in RDW^43^.

Diabetes mellitus plays a pivotal role in recurrent atherothrombotic events, especially in patients with acute coronary syndrome (ACS) that underwent percutaneous coronary intervention (PCI)^44^ due to risk factors including hyperglycemia, insulin deficiency, and metabolic dysregulation resulting in platelet dysfunction^45^. In addition, “prolonged” antiplatelet therapy could decrease vascular events by approximately 1/4 in diabetic and non-diabetic subjects^46^. However, antiplatelet therapy may be associated with the elevation of RDW. Here, we found that antiplatelet therapy, such as aspirin or clopidogrel, showed a higher proportion in Q2 or Q4 of RDW in T2D patients (Table 3 and Fig. 3a), suggesting these diabetic patients, characterized by chronic inflammation and oxidative conditions, might experience rearrangement of the cytoskeleton and loss of asymmetric lipids of the RBC47, resulting in high levels of RDW.

In conclusion, our results provide novel insight into the relationship between RDW and basic characteristics, blood and urine examinations, complications and medications in DM. First, a graded association between disease duration and RDW was obtained, showing that longer durations were correlated with increasing RDW, and that older patients had significantly higher RDW levels than younger patients. Second, serum Ca^2+^ and Hb may be protective in decreasing the level of RDW. Third, an increased level of RDW was accompanied by elevated levels of serum Cr and proteinuria in type 2 diabetes patients. Finally, the elevated level of RDW with antiplatelet therapy indicates that RDW might be a new biomarker for evaluating the dosage of antiplatelet drugs. However, several limitations should be mentioned in our study. For example, the present study is a cross-sectional study, and the relationship between RDW and other indexes is temporal or casual. Altogether, RDW may be a significant and accessible biomarker in T2D patients relative to clinical detection and evaluation.

## Materials and Methods

### Patients and study design

At the beginning of this trail, we obtained informed consents from subjects and received approval from the Human Research Ethics Committees in Second Xiangya Hospital of Central South University (approval number: 2015-SO26). The study was performed in accordance with the approved guidelines. Written informed consent was obtained from all patients enrolled in the study. 809 patients were diagnosed with type 2 diabetic disease in the Endocrinology Department of Second Xiangya Hospital of Central South University from June 2014 to November 2015. According to the statistical quartiles method of RDW (%), 809 patients were classified as Q1 ≤ 12.4 (229 patients), Q2 > 12.4 and ≤ 12.9 (202 patients), Q3 > 12.9 and < 13.5 (168 patients), or Q4 ≥ 13.5 (210 patients). Then, the association between RDW and diabetes was measured by comparing group Q1 with groups Q2, Q3 and Q4. The exclusion criteria include the following: type 1 diabetic patients or latent autoimmune diabetes in adults (LADA), patients with known inflammatory-related diseases such as rheumatoid arthritis and systematic lupus erythematosus, and pregnant diabetic women. The diagnostic criteria for type 2 diabetes were defined as follows: HbA1c level ≥6.5%; or fasting plasma glucose level ≥126 mg/dL (≥7.0 mmol/L); or 2-h plasma glucose level ≥200 mg/dL (≥11.1 mmol/L) in an oral glucose tolerance test (OGTT); or a casual plasma glucose level of ≥200 mg/dL (≥11.1 mmol/L)^47^. Data of demographic characteristics in diabetic patients including age, sex, and smoking and drinking habits were collected. Smoking or drinking daily for at least 1 year classified patients as smokers or drinkers, respectively. The formula for BMI was calculated as weight (kg)/height [m^2^]. DR was diagnosed with varying degrees of microaneurysms, hemorrhages, exudates, change of vein, new vessel formation and retinal thickening consisting of background (mild non-proliferative), preproliferative (moderate/severe non-proliferative), proliferative and advanced retinopathy. Involvement of the macula can be foca, diffuse, ischemic or mixed. The criteria for DPN were mainly based on clinical symptoms (such as sensation decreases, neuropathic-related sensory symptoms), neurologic examination and electrophysiologic investigation. The definition of HTN was based on systolic blood pressures ≥140 mmHg and/or diastolic blood pressures ≥90 mmHg measured three different but consecutive times on the condition that patients had not used antihypertensive drugs. CHD was defined as myocardial impairment due to an imbalance between coronary blood flow and myocardial requirements caused by changes in the coronary circulation, which was subdivided into acute coronary syndrome and chronic coronary artery syndrome. Dysglycemia was characterized by either fasting plasma glucose (FPG) ≥5.6 mmol/L or HbA1c ≥5.7%. Oral drug use was defined as the patients taking medications regularly for 3 months.

### Laboratory examination

Hematologic testing was performed with the ADVIA 2120 automated hematology analyzer (Siemens Healthcare Diagnostics, Germany), measuring hemoglobin, white cell count, RDW, mean platelet volume, platelet crit, platelet large cell ratio, and platelet distribution width, as described previously^48^. The liver and renal function parameters, including BUN, Cr, UA, Alb, TG, TC, HDL, LDL and FBG levels, were analyzed using standard automated enzymatic methods (Hitachi 912 automated analyzer), as previously described^49^. Other biochemical examinations including CRP, serum Ca^2+^, and P were detected on the C8000 Abbott ARCHITECT Clinical Chemistry Analyzers (Abbott Diagnostics, USA). In addition, proteinuria was defined as a urine albumin excretion rate (UAER) of greater than 30 mg/24 h^50^. Urine concentrations of albumin were measured by the immunoturbidimetric method as described previously^48, 49^. In addition, HbA1c evaluations were performed with automatic high-performance liquid chromatography (HPLC) (VARIANT-II Hemoglobin Testing System; Bio-Rad Laboratories, Hercules, CA)^51^.

### Statistical analysis

Quantitative (such as Hb, SBP, DBP, Upro) and qualitative (such as sex, smoking) variables are described as the mean ± standard deviation and percentages, respectively, and the differences between groups Q4 and Q1 were compared using the T-test or the χ^2^ test or by the non-parametric Kruskal-Wallis test when the *P* value of the 1-sample Shapiro-Wilk test <0.05. Then, the significant variables were used to evaluate the risks associated with RDW by the Logistic regression method. For the requirement of Logistic regression, these quantitative variables were classified as positive or negative by a cut off value, which is antiplatelet drugs: “1” refers to patients not using antiplatelet drugs, “2” refers to patients using antiplatelet drugs; HTN: “1” refers to patients with DM alone, “2” refers to patients with DM and HTN. Age (years): “1” refers to “<51”, “2” refers to “≥51” but “<60”, “3” refers to “≥60” but “<68”, and “4” refers to “≥68”; Hb (g/dL): “1” refers to “60–90”, “2” refers to “91–109”, and “3” refers to “≥110”, which is consistent with the clinical classification. Serum Cr (µmmol/L): “1” refers to “<133”, “2” refers to “≥133” consistent with the critical value in our hospital. Serum Ca^2+^ (mmol/L): “1” refers to “<2.03”, “2” refers to “≥2.03” but “≤2.54”, and “3” refers to “>2.54”; urinary protein excretion (Upro): “1” was defined as proteinuria less than 30 mg/L, and “2” was defined as proteinuria more than 30 mg/L; Duration (years): “1” refers to “<3”, “2” refers to “≥3” but “<9”, “3” refers to “≥9” but “<14”, and “4” refers to “≥14”. The data were analyzed using *SPSS 19.0* (SPSS Inc, USA), and *P* value of <0.05 was regarded as statistically significant.
